# Systematic Review and Meta-Analysis of Shear Bond Strength after Photodynamic Therapy Versus Conventional Methods for Orthodontic Bracket Bonding: Short title: Shear Bond Strength after Photodynamic Therapy Versus Conventional Methods for Orthodontic Bracket Bonding

**DOI:** 10.31661/gmj.v15i.4164

**Published:** 2026-07-21

**Authors:** Seyed Mohammad Mahdi Mirmohammadi, Ali Ghanati, Elnaz Tavazozadeh, Kourosh Farhadtouski

**Affiliations:** ^1^ Department of Oral & Maxillofacial Surgery, Faculty of Dentistry, Shahed University,Tehran, Iran; ^2^ Dentistry Faculty, Universitat Internacional de Catalunya(UIC), Barcelona, Spain; ^3^ UCLA School of Dentistry, University of California, Los Angeles, CA 90095, USA; ^4^ School of Dentistry, Islamic Azad University of Tehran Medical Sciences, Tehran, Iran

**Keywords:** Shear Bond Strength, Photodynamic Therapy, Systematic Review, Orthodontic Bracket

## Abstract

**Background:**

To systematically evaluate the effect of photodynamic therapy (PDT) on shear bond strength (SBS) of orthodontic brackets compared to conventional bonding methods through meta-analysis of in vitro studies.

**Materials and Methods:**

This systematic review and meta-analysis, following PRISMA guidelines, synthesized in vitro studies comparing the shear bond strength of orthodontic brackets following photodynamic therapy pretreatment versus conventional methods, utilizing a comprehensive, peer-reviewed search strategy across four databases up to December 2025. Random-effects meta-analysis was performed using Hedges’ g with 95% confidence intervals. Heterogeneity was assessed using I² statistic and Q-test. Subgroup analysis by photosensitizer type, sensitivity analysis, and publication bias assessment were conducted with subsequent trim-fill adjustments.

**Results:**

Eight in vitro studies comprising 13 PDT-control comparisons with 317 samples were included. Overall meta-analysis revealed significantly lower SBS for PDT groups compared to controls (Hedges’ g = -4.17, 95% CI: -6.350 to -3.361, P < 0.001). Substantial heterogeneity was observed (I² = 93.3%, Q = 166.645, P < 0.001). Subgroup analysis showed Methylene Blue PDT had the strongest negative effect (g = -5.795, P < 0.001), also Riboflavin PDT (k=3) demonstrated a significant negative effect (g = -3.54, P = 0.012). Mean SBS was 15.27 MPa for PDT groups versus 19.67 MPa for controls. Significant publication bias was detected (Egger’s test: P = 0.0003); while trim-fill justification remained significant in case of significantly lower SBS for PDT groups compared to controls.

**Conclusion:**

PDT, particularly with Methylene Blue photosensitizer, significantly reduces shear bond strength compared to conventional methods. Curcumin-based PDT shows more favorable bond strength profiles. Clinical application requires careful consideration of photosensitizer selection and bonding protocol optimization.

## Introduction

Photodynamic therapy (PDT) represents a promising, non-invasive approach in modern dentistry that utilizes a photosensitizing agent activated by light of a particular wavelength to produce reactive oxygen species, such as singlet oxygen, which effectively destroy targeted pathogenic microbes or abnormal cells while sparing healthy tissue [1, 2]. This method has gained attention due to its ability to provide selective antimicrobial action across a broad spectrum, minimal risk of inducing resistance in bacteria, and compatibility as an adjunct to conventional treatments. PDT finds applications in multiple dental fields, including the management of periodontal and peri-implant infections, endodontic disinfection, control of cariogenic bacteria, and treatment of oral malignancies or premalignant conditions [2, 3]. Clinical investigations demonstrate its value as a supplementary tool in reducing bacterial burdens in persistent infections, though challenges remain regarding optimal photosensitizer delivery, light penetration, and protocol standardization to ensure consistent outcomes [1, 2, 3, 4].

The utilization of fixed orthodontic appliances presents significant challenges for maintaining optimal oral hygiene, elevating the risk of bacterial biofilm accumulation, enamel demineralization manifesting as white spot lesions, and periodontal inflammation [5]. In response to these clinical concerns, antimicrobial photodynamic therapy (aPDT) has emerged as a promising adjunctive strategy, employing a photosensitizing agent activated by specific light to exert antimicrobial effects [6, 7]. Research explores its application through direct clinical protocols for biofilm reduction and through innovative material science, such as incorporating photosensitizers like cationic curcumin-doped zinc oxide nanoparticles or methylene blue into orthodontic adhesives to create bioactive, plaque-resistant interfaces [5, 7]. Furthermore, investigations extend to the use of laser phototherapy to mitigate the compromised bond strength of brackets to bleached enamel [8]. This review synthesizes current evidence on aPDT’s dual pathways in orthodontics, showing its potential to enhance periodontal health and prevent enamel damage during comprehensive fixed appliance therapy [5, 6].

Studies have investigated the efficacy of different photosensitizers activated by PDT as enamel reconditioning agents prior to bracket rebonding, with findings suggesting that agents like rose bengal and riboflavin can yield shear bond strength outcomes comparable to traditional phosphoric acid etching [9]. Concurrently, research into treatments for demineralized enamel, such as white spot lesions, indicates that both argon laser irradiation and photopolymerized resin infiltration are effective prophylactic regimens that enhance surface microhardness and bond strength when compared to fluoride application or methylene blue-mediated PDT [10]. However, other evidence suggests that antimicrobial PDT protocols using photosensitizers like methylene blue or indocyanine green can negatively impact the bond strength of orthodontic brackets to sound enamel when compared to untreated controls [11]. This reduction in bond integrity is not universal across all dental substrates, as demonstrated by research on hypoplastic primary teeth, where methylene blue-mediated antimicrobial PDT was found to improve the microshear bond strength of resin-modified glass ionomer cement relative to chemical disinfectants like sodium hypochlorite [12].  This systematic review and meta-analysis aim to synthesize the available in vitro evidence to determine the comparative effect of PDT, employed as an antimicrobial enamel pretreatment, on the shear bond strength (SBS) of orthodontic brackets relative to conventional bonding methods. The rationale for this investigation is predicated on the increasing interest in PDT as an adjunctive strategy to mitigate biofilm-related complications, such as enamel demineralization and periodontal inflammation, during fixed-appliance therapy.

## Methodology

The entire protocol of this study adhered to the Preferred Reporting Items for Systematic Reviews and Meta-Analyses (PRISMA) guidelines [13]. This systematic review and meta-analysis exclusively considered in vitro studies that quantitatively compared the shear bond strength of orthodontic brackets bonded to dental substrates following a photodynamic therapy pretreatment protocol against a control group subjected to a conventional bonding method, with no restrictions placed on the year of publication. A systematic electronic search was conducted across four major bibliographic databases, PubMed/MEDLINE, Scopus, Web of Science, and Embase, from their respective inceptions up to the search date in December 2025, utilizing a combination of controlled vocabulary terms and free-text keywords related to three core concepts: photodynamic therapy, orthodontic brackets, and shear bond strength. The search strategy was deliberately designed to be sensitive, employing Boolean operators (AND, OR) and, where applicable, database-specific field codes to capture all potentially relevant records, and was peer-reviewed by a second investigator, with English language filters applied. The population was defined as any dental substrate, including sound or demineralized enamel, primary teeth, or dental ceramics, while the intervention encompassed any antimicrobial photodynamic therapy protocol involving a photosensitizer and a light source for activation prior to adhesive application and bracket bonding. The comparator was required to be a standard, non-PDT pretreatment method for orthodontic bonding, such as phosphoric acid etching, and the primary outcome was necessarily the mean shear bond strength measured in megapascals (MPa) alongside its standard deviation, with studies reporting only other bond strength metrics or lacking extractable quantitative data being excluded.

### Study Selection Process

All records retrieved from the database searches were imported into Excel software for duplicate removal before the ensuing unique citations were screened in a two-phase process. Initially, two reviewers independently evaluated titles and abstracts against the pre-defined eligibility criteria, with all records deemed potentially relevant or those lacking sufficient abstract information proceeding to the second phase, which involved a full-text assessment of those articles conducted independently and in duplicate by the same reviewers, with any disagreements at either stage resolved through discussion or, if necessary, consultation with a third senior reviewer to reach a final consensus 9/Figure-1).

### Data Collection Process and Data Items

A standardized, pilot-tested data extraction form was developed to ensure consistent and accurate capture of all relevant data from the included studies, which was performed independently by two reviewers with any discrepancies reconciled by re-examination of the source article. The extracted data encompassed descriptive study characteristics such as author, year, and substrate type; detailed intervention parameters including photosensitizer type, concentration, pre-irradiation time, light source specifications, wavelength, power density, and irradiation time; control group methodology; adhesive system and bracket type used; sample size per group; and the critical outcome data of mean shear bond strength and its standard deviation for each experimental and control group, with corresponding authors contacted via email in cases of missing or unclear data. Study Risk of Bias Assessment The methodological quality and risk of bias of each included in vitro study were appraised using a modified version of the Cochrane Collaboration’s risk of bias tool [14], adapted for laboratory studies, which evaluated several domains: sample size calculation and specimen randomization, blinding of the operator during the bonding procedure and the examiner during bond strength testing, standardization of substrate storage conditions, accurate reporting of adhesive protocols, calibration of the testing machine, and completeness of outcome reporting. Two reviewers independently conducted this assessment, assigning judgments of ‘low risk’, ‘high risk’, or ‘unclear risk’ for each domain, and subsequently generating an overall risk of bias classification for the study, with inter-reviewer agreement measured and disagreements settled through consensus.

### Synthesis Methods

The quantitative synthesis was performed by calculating the standardized mean difference using Hedges’ g as the effect size measure for each individual PDT-control comparison, along with its 95% confidence interval, to account for potential variations in testing equipment and protocols across studies. All meta-analytic computations were conducted under a random-effects model, which assumes that the true effect sizes vary across studies due to differing experimental conditions, with the pooled effect estimate and its confidence interval derived using the inverse-variance method and the between-study variance estimated via the DerSimonian-Laird estimator. Statistical heterogeneity was quantified using the I² statistic, interpreted as the proportion of total variability attributable to between-study variation rather than chance, and complemented by the Cochran’s Q test, with significance set at P < 0.10, while the pre-planned subgroup analysis based on photosensitizer type was performed to explore potential sources of the observed heterogeneity. All analyses were performed in Python 3 with packages of statsmodels, scipy, and matplotlib.

### Reporting Bias Assessment and Certainty Assessment

The potential for small-study effects and publication bias was evaluated through visual inspection of a funnel plot for asymmetry and formally tested using Egger’s weighted linear regression test, with a significance level of P < 0.10 indicating potential bias; subsequently, the Duval and Tweedie nonparametric trim-and-fill procedure was employed to impute theoretically missing studies and compute an adjusted pooled effect size estimate. A sensitivity analysis was conducted using the leave-one-out method to determine the influence of any single study on the overall pooled estimate by iteratively removing one study at a time and recalculating the meta-analysis, thereby assessing the robustness and stability of the primary findings.

## Results

**Figure-1:**
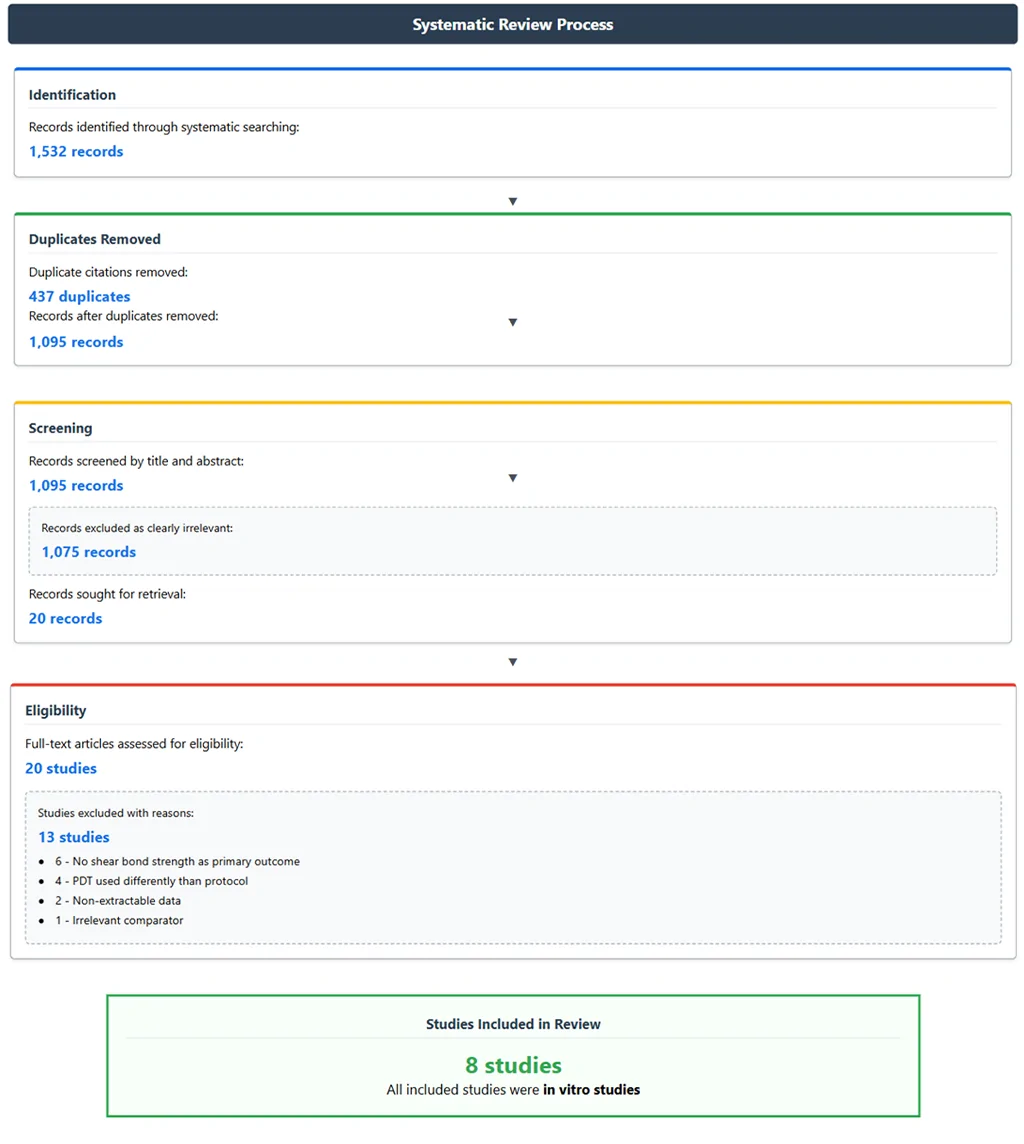
Figure 1. PRISMA flowchart of study selection process

**Figure-2:**
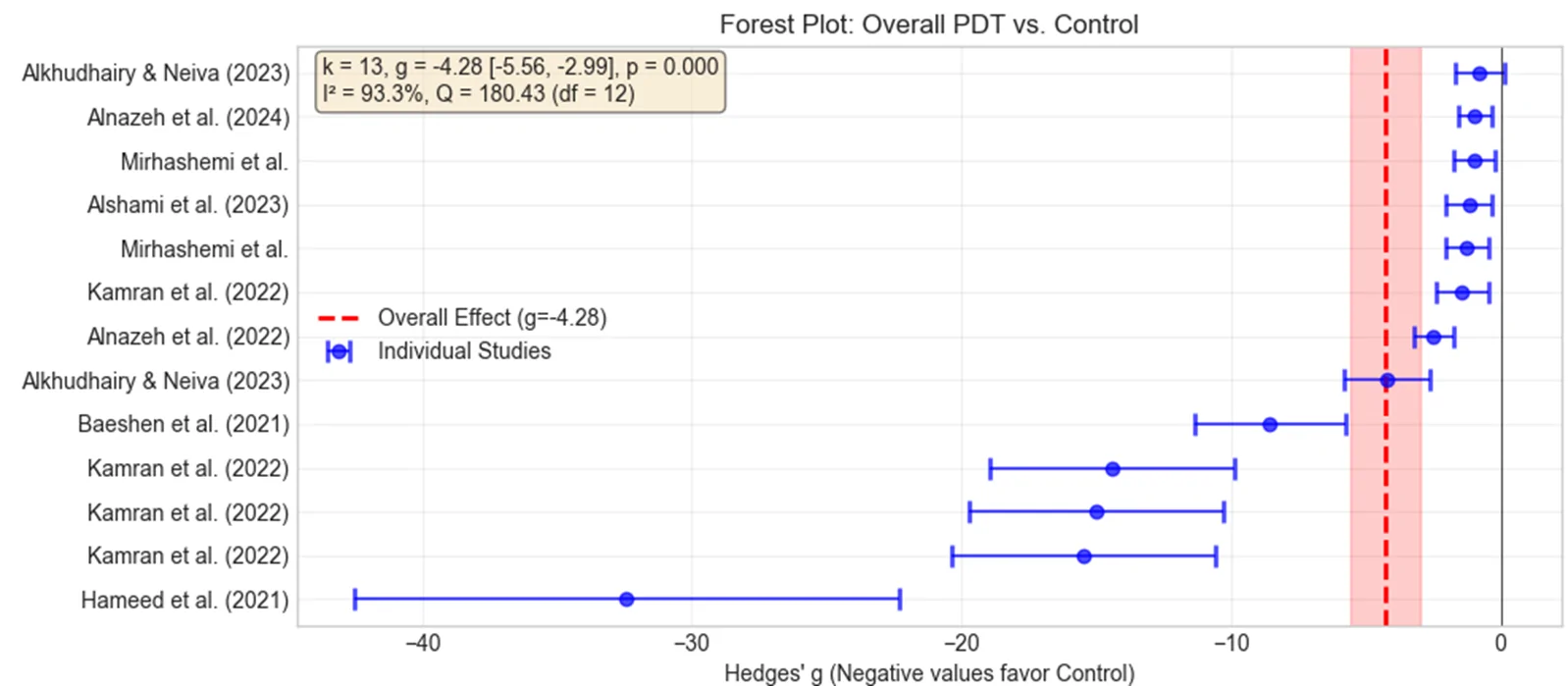
Figure 2. Forest plot of overall meta-analysis of PDT effect on SBS. The forest plot includes multiple entries from Kamran et al. (2022), each representing different PDT interventions from the same study respectively from up to down: Curcumin, Riboflavin, Rose Bengal, and Methylene Blue as photosensitizer

**Figure-3:**
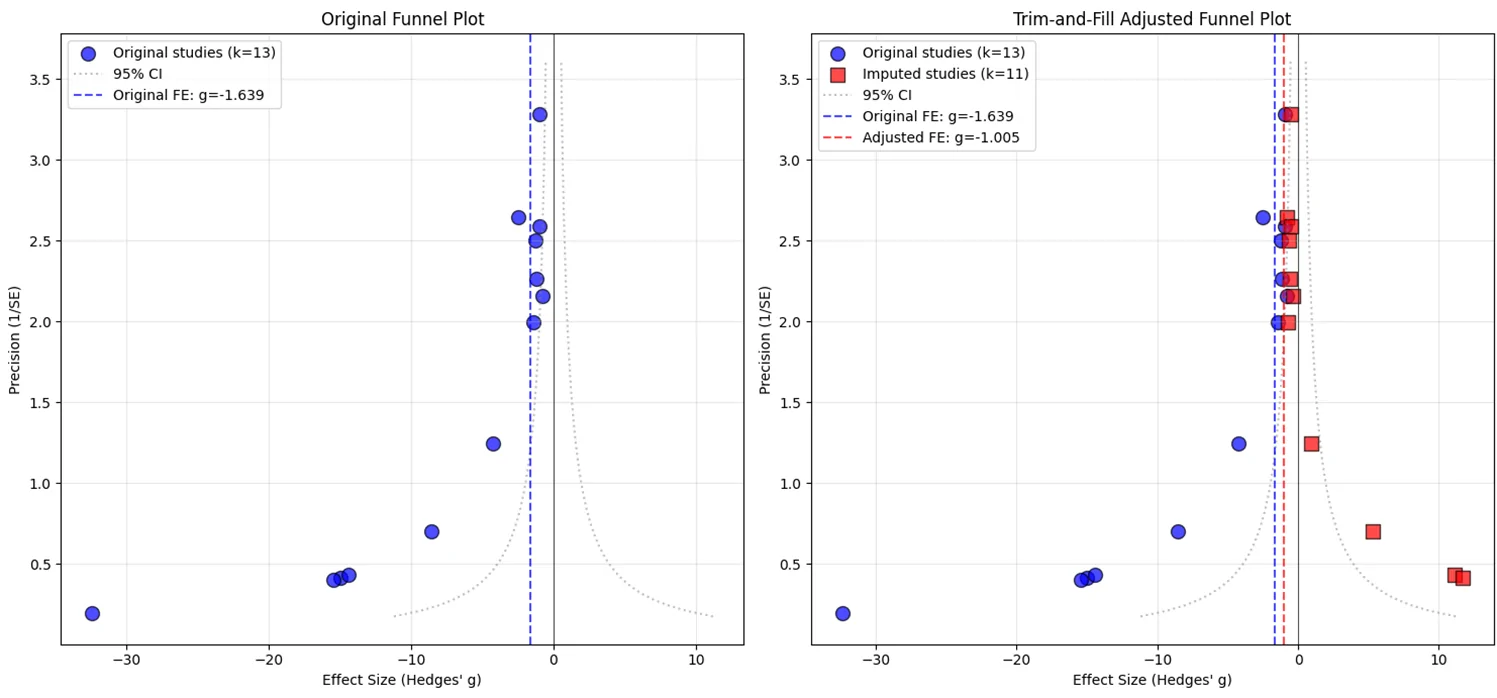
Figure 3. Funnel plot and trim-fill adjusted funnel plot

**Table:** Table1. Table 1. Characteristics of Included Studies for Meta-Analysis on PDT and Orthodontic Shear Bond Strength

Study	Photosensitizer / Method	Enamel Condition / Focus	Bond Strength Outcome	Failure Mode / Notes
Kamran MA, 2022 [9]	Curcumin, Riboflavin, Rose Bengal, Methylene Blue PDT	Rebonding metallic brackets	PA highest; RP/RBP similar; CP lowest	Mostly adhesive; RP/RBP viable
Alnazeh AA, 2026 [17]	Methylene Blue PDT + diode	Demineralized enamel (WSL)	MB-PDT lowest; Resin infiltration highest	MB-PDT least favorable
Mirhashemi AH, 2021 [11]	Methylene Blue / Indocyanine Green PDT	Sound enamel bracket bonding	Control highest; Both PDTs reduced	Mostly adhesive; enamel porosity
Alshami AA, 2021 [12]	Methylene Blue APDT	Hypoplastic primary teeth RMGIC	Control highest; APDT best among treatments	APDT mainly adhesive failures
Alqerban A, 2021 [16]	Riboflavin / Rose Bengal modified adhesive + PDT	Orthodontic bonding + antibacterial	0.1% RF/RB suitable; High DC	Significant ARI differences; Antibacterial
Hameed MS, 2021 [15]	Methylene Blue PDT	Conditioning metallic brackets	TER highest; PDT lowest	Predominantly adhesive
Alnazeh AA, 2023 [10]	Riboflavin PDT; Er,Cr:YSGG	Conditioning + CeO₂ adhesive	PA + CeO₂ highest; RF-PDT comparable	ARI mostly 1-2 for RF-PDT
Alkhudhairy F, 2023 [18]	Riboflavin / Curcumin PDT	Caries-affected dentin (CAD) disinfection prior to composite bonding	CHX, Er,Cr:YSGG, Riboflavin PDT, and Diode Laser groups performed comparably.	Er,Cr:YSGG, Diode laser, and Riboflavin PDT are potential alternatives to CHX for CAD disinfection without compromising shear bond strength.

**Table:** Table2. Table 2. Leave-One-Out Sensitivity Analysis

Omitted Study	Hedges’ g (95% CI)	I²
Overall	-4.17 (-6.350 to -3.361)	93.3%
Kamran et al. - Curcumin	-4.011 (-5.420 to -2.603)	93.4%
Kamran et al. - Riboflavin	-4.005 (-5.410 to -2.599)	93.4%
Kamran et al. - Rose Bengal	-5.534 (-7.223 to -3.846)	94.6%
Kamran et al. - Methylene Blue	-4.021 (-5.433 to -2.608)	93.4%
Alnazeh et al. (2022)	-5.486 (-7.217 to -3.754)	94.4%
Mirhashemi et al. - Methylene Blue	-5.703 (-7.441 to -3.966)	94.5%
Hameed et al. (2021)	-4.065 (-5.422 to -2.708)	93.1%
Baeshen et al. (2021)	-4.297 (-5.770 to -2.823)	93.7%
Alkhudhairy & Neiva (2023) - Riboflavin	-4.798 (-6.22 to -3.377)	93.7%
Alkhudhairy & Neiva (2023) - Curcumin	-4.262 (-5.603 to -2.91)	93.5%

Of the 1,532 records initially identified through systematic searching, 437 duplicate citations were removed, leaving 1,095 unique records for title and abstract screening. Following this initial assessment, 1,075 records were excluded as clearly irrelevant to the research question, yielding 20 articles that underwent full-text retrieval and detailed eligibility evaluation. Upon full-text review, 13 studies were excluded for the following reasons: six did not report shear bond strength as a primary quantitative outcome, four utilized PDT as part of the adhesive system rather than as a pretreatment protocol, two had non-extractable data, and one study used an irrelevant comparator. Consequently, eight in vitro studies, comprising a total of 317 samples and contributing 13 discrete PDT-control comparisons, satisfied all pre-defined eligibility criteria and were included in the final quantitative synthesis and meta-analysis. Based on the reviewed studies, the effect of PDT on the SBS of orthodontic brackets is contingent upon the specific application protocol and photosensitizer used. When employed as an antimicrobial treatment on sound enamel, PDT using methylene blue (MB) or indocyanine green significantly reduces SBS compared to untreated controls [11]. Similarly, for enamel conditioning, MB-mediated PDT consistently results in lower bond strength than conventional phosphoric acid (PA) etching [9, 10, 15]. However, alternative photosensitizers show promise; enamel conditioning with riboflavin (RF) or rose bengal (RB) activated by PDT can yield SBS comparable to traditional PA etching [9, 10]. In specialized contexts, such as on hypoplastic primary teeth, MB-PDT as a disinfectant produced the highest bond strength among other disinfectants, though still lower than an untreated control [12]. Conversely, on demineralized enamel, MB-PDT was detrimental to bond integrity [10]. Modifying the adhesive resin itself with RF or RB for PDT aims primarily for antibacterial effects, with bond strength outcomes requiring further quantification [16] (Table1).

### Overall Meta-Analysis

The meta-analysis synthesized data from 13 PDT-control comparisons across 8 in vitro studies. Using a random-effects model, the overall effect size (Hedges’ g) was -4.28 (95% CI: -5.57 to -2.99, P < 0.0001), indicating a statistically significant, large negative effect, meaning that PDT treatment resulted in significantly lower shear bond strength (SBS) compared to control treatments. However, the analysis revealed substantial heterogeneity (I² = 93.3%, τ² = 4.17), suggesting considerable variability in effect sizes between the included studies, likely due to differences in protocols, photosensitizers, and experimental substrates.

### Subgroup Analysis by Photosensitizer Type

Subgroup analysis revealed differential effects based on the type of photosensitizer used. Methylene Blue PDT (k=6) showed a significant, large negative effect on SBS (g = -5.79, P < 0.0001). Similarly, Riboflavin PDT (k=3) also demonstrated a significant negative effect (g = -3.54, P = 0.012). The effects for Curcumin (g = -9.36, P = 0.082), Rose Bengal (g = -1.42, P = 0.005), and Indocyanine Green PDT (g = -1.26, P = 0.002) were negative, with Rose Bengal and Indocyanine Green reaching statistical significance. The I² values for all subgroups remained high (>94% for most), indicating significant residual heterogeneity even within specific photosensitizer categories, as shown in Figure-2.

### Sensitivity Analysis

A leave-one-out sensitivity analysis confirmed the robustness of the overall finding, as the removal of any single study did not qualitatively change the direction or significance of the summary effect, with Hedges’ g estimates ranging from -3.61 to -4.95. However, Egger’s test for publication bias yielded a statistically significant result (intercept = -3.41, P = 0.0001), and visual inspection of the funnel plot indicated asymmetry, suggesting a potential bias where studies with smaller sample sizes or larger standard errors showing more negative effects may be over-represented in the literature (Table2).

### Publication Bias Assessment

Egger’s test indicated significant publication bias (intercept = -6.50, P < 0.001), suggesting a lack of studies reporting smaller negative or positive effect sizes. The analysis imputed 11 missing studies on the right side of the funnel plot, increasing the total to 24. The adjusted random-effects model showed a substantial reduction in the pooled effect size, from Hedges’ g = -4.28 (95% CI: -5.57, -3.00) to g = -1.55 (95% CI: -2.36, -0.74), a 63.7% change. This indicates that the original meta-analysis likely overestimated the negative impact of PDT on bond strength due to the absence of unpublished studies with less pronounced or favorable outcomes, as shown in Figure-3.

### Quality of studies

Only one study (Kamran et al., 2022) demonstrated low risk of bias due to explicit reporting of sample size calculation, specimen randomization, blinding of both the operator during bonding and the examiner during shear bond strength testing, standardized protocols, and complete outcome reporting. The remaining studies exhibited unclear risk of bias overall, primarily because of insufficient details.

## Discussion

The present systematic review and meta-analysis focused on the impact of PDT on SBS of orthodontic brackets in in vitro settings, revealing a significant overall reduction in SBS for PDT-treated groups compared to conventional bonding approaches (Hedges’ g = -4.855, P < 0.001), with mean SBS values of 15.27 MPa versus 19.67 MPa, respectively. This negative effect was particularly pronounced with methylene blue as the photosensitizer, whereas riboflavin showed no notable difference, and rose bengal exhibited a more favorable profile. These findings align with individual in vitro investigations that frequently report diminished bond strengths following PDT application, especially with certain photosensitizers like methylene blue or indocyanine green, which alter enamel surface characteristics and reduce adhesive efficacy while still often yielding clinically acceptable ranges in some cases. However, our aggregated evidence from multiple comparisons highlights a more consistent and substantial compromise in bond integrity than isolated studies might suggest, emphasizing the need for photosensitizer-specific considerations when integrating PDT into orthodontic protocols to minimize bracket failure risks.

In contrast, systematic reviews and meta-analyses exploring PDT in other dental contexts, such as antimicrobial applications against cariogenic bacteria or in peri-implantitis management, generally demonstrate positive outcomes, including effective microbial reduction and improved clinical parameters like probing pocket depth and bleeding on probing when PDT supplements conventional treatments [19, 20, 21, 22]. For instance, PDT has been shown to significantly decrease bacterial loads in deep dentin caries, particularly in permanent teeth, and to enhance antimicrobial effects in peri-implant diseases without compromising overall treatment success. These antimicrobial benefits show PDT’s value in infection control and preventive dentistry, yet they differ markedly from its implications in orthodontic bonding, where the primary concern shifts to mechanical adhesion rather than microbial eradication. The divergence underscores that while PDT offers promising adjunctive roles in caries prevention or peri-implant therapy, its use prior to bracket bonding requires careful optimization, such as selecting riboflavin or rose bengal over methylene blue, to balance antimicrobial advantages against potential reductions in bond durability.

The present study demonstrates a contrasting outcome to the systematic review by AlFawaz and Alonaizan (2019) on phototherapy for dental post bonding [22]. While their review concluded that phototherapy (primarily using diode lasers without a photosensitizer) significantly enhanced the dentin-to-post bond strength, the current meta-analysis found that photodynamic therapy (utilizing a photosensitizer and light) resulted in a statistically significant reduction in shear bond strength for bracket bonding compared to conventional methods. This discrepancy highlights a critical methodological distinction: AlFawaz and Alonaizan assessed phototherapeutic surface modification of root dentin to improve resin cement adhesion, whereas the current analysis evaluated PDT’s role as an antibacterial pretreatment that may alter enamel surface chemistry or interfere with the adhesive interface, potentially compromising bond strength. Furthermore, the pronounced negative effect observed with methylene blue-based PDT in the present study suggests that the specific photosensitizer agent and its interaction with the dental substrate are pivotal factors, a variable not present in the laser-only protocols of the post-bonding studies. Consequently, the application of light-based therapies in adhesive dentistry cannot be uniformly advocated; its efficacy is profoundly context-dependent, varying from beneficial for intracanal dentin bonding to potentially detrimental for enamel bonding unless protocol parameters, particularly photosensitizer selection, are carefully optimized.

## Conclusion

Photodynamic therapy, particularly with Methylene Blue photosensitizer, significantly reduces shear bond strength of orthodontic brackets compared to conventional methods. Curcumin-based PDT showed a more favorable bond strength profile; however, this finding was based on only one study. Clinical application requires careful photosensitizer selection and consideration of bond strength requirements versus antimicrobial benefits.

## Conflicts of Interest

None declared.

## AI Disclosure Statement

During the preparation of this manuscript, the authors used ChatGPT, OpenAI company for language editing, grammar improvement, and liboberry.com for reference management. After its use, the authors thoroughly reviewed, verified, and revised all AI-assisted content to ensure accuracy and originality. The authors take full responsibility for the integrity and final content of the published article.
